# Medicated Squab Pie

**Published:** 1910-12-03

**Authors:** A. H. Bampton


### THE GENERAL PRACTITIONER'S COLUMN.

[Contributions to this Column are invited, and if accepted, will be paid for.]

### MEDICATED SQUAB PIE.

By A. H. BAMPTON, M.D., M.Ch., M.O.H., Ilkley.*

GENTLEMEN,—The mind of your Chairman must always be vexed and troubled to find a suitable subject upon which to address you, the more especially because at this season of the year the flood-gates of eloquence are opened to all and sundry, both qualified and unqualified, from generals to authors, descanting on the merits and the demerits of the medical profession in the most appreciative and sympathetic terms. What concerns me thereby is that all popular subjects become worn threadbare and hackneyed, and I am thrown back upon personal experiences, which form of speech is so characteristic of a garrulous old age. After all, personal experiences are more interesting than rehashed text-book truisms or mountains of theory erected on a grain of fact.

I entered the medical profession some thirty-six years ago through the old-fashioned portal of medical pupilage. I do not altogether regret the time thus spent, as it enabled one to obtain a first-hand knowledge and acquaintance with the dispensing and use of remedies: old-fashioned maybe, but still amongst the most trusted and certain weapons in the healer's armamentarium. Many of the new remedies are but the rediscovery of the old, *e.g.* calcium salts, senna pods, and phosphoric acid. Is there any combination of remedies that give more gratifying results in heart disease with dropsy than old Budd's—of Barnstaple—mixture of digitalis and iron? Unsightly, unpleasant, incompatible, yet efficient!

Reminiscences of medical practice have this in common with golfing episodes, that one remembers, or recounts only, the "good shots" that one has made on the round. Mercifully, the record is but a faint one that tells of our mistakes. In these days of slavish adherence to strict aseptic surgery one marvels how in former times we ever got anything to heal. I remember the first operation for fistula in recto I did, and marvel at my temerity. I had been called nine miles into the country to see the case in a hurry, and found the patient in great pain and demanding immediate relief. I had been told nothing of the case, and was totally unprovided with suitable instruments. Evening was closing in, and if I rode back it would mean thirty-six miles for one case. I had in my pocket-case knife and director, and with a wax candle in the rectum as protection, I cut down upon the fistula. In spite of this crude surgery the patient did well! It is easy to criticise rough and ready methods when one has at hand all the resources of modern surgical civilisation. On another occasion I was called in to see a woman in a comatose condition from retention of urine. I found an enormously distended bladder, mechanically obstructed from malignant growth in the pelvis. I had no catheter, the case was urgent, so I sent round to the village inn for a churchwarden pipe, and passed the stem into the bladder; as the bladder emptied the patient recovered consciousness, and realising the situation showed marked signs of bewilderment, thinking, no doubt, that the world had gone topsy-turvey.

This incident reminds me of another. A practitioner who was called on his round to a gipsy encampment found a woman in labour, delayed by a distended bladder; having no catheter, he bethought of a stout dandelion stem, which is hollow, and with this he successfully relieved the poor woman's bladder, when the labour speedily came to an end.

One is apt to forget that in rural districts, where the subjects are healthy and the air pure, excellent results were obtained in surgery by methods of ordinary cleanliness. I remember attending at Redruth, in the early eighties, a divisional meeting of the British Medical Association, when the late Sir W. MacCormac gave an address on two cases of cerebral surgery which he had had in St. Thomas's Hospital. At the conclusion of his paper the local practitioners trooped in some thirty or forty patients, all recovered from most extensive head injuries received in that mining district and treated by the simple methods of the village surgeon. Sir. Wm. MacCormac was much amused, and acknowledged that he had brought coals to Newcastle. On another occasion I was hastily summoned one early morning to see a patient who had swallowed her false teeth. She told me that she had dreamt that I had ordered her to take a large bolus, and in the effort of attempting to swallow it, awoke to find that the bolus was her false teeth. When I got there the plate was in the lower part of the œsophagus, and I thought it was dangerous to attempt to drag back a plate with sharp edges. So I adopted the expectant method, waiting on Providence. No urgent symptoms arose, and the patient could tell me from day to day in what portion of her abdomen the teeth were. The journey through the gut took two months to complete, when the teeth and plate were passed in good condition. At the time of the misadventure the patient was suffering from pleurisy, which, curiously enough, speedily took a turn for the better; she credits the gold plate with the cure.

I must apologise if I have wearied you with these commonplace experiences, and would conclude with a political note on medical economics. A subject that I have had to heart for many years is coming to the front for settlement. Some years since a writer addressed us on the "Political Powerlessness of the Medical Profession." I would emphasise and explain this position by dwelling on the pecuniary powerlessness of the medical profession. We are politically powerless because we are pecuniarily powderless. Money is power. And we

* Substance of an Address given as Chairman of the Bradford Division of the British Medical Association.

are poor because we do not get the just fruits of our labour. As a profession we are exploited by Parliament and the public, and bear an undue share of the burden of charity. Has it ever been computed what is the value of the medical and surgical services rendered in the free hospitals of the United Kingdom? I wish Mr. Loch would undertake the computation and make it known. I am sure that in many of our general hospitals there are surgeons whose services yearly, computed on the lowest Poor-Law scale of fees, are valued at not less than £10,000. Now if a layman gives £10,000 in one sum to an institution he is knighted at once, and has a memorial erected to his memory. But the surgeon, it is said, "He has the experience as a full equivalent to the services rendered." "Justice" is a holy thing and a national requirement. Let us commemorate the next national event by the erection of "Halls of Justice" all over the kingdom, wherein, for the love of justice and equity, the judge, barristers, and solicitors, elected and patronised by a lay committee, shall practise daily without fee or salary. Why not? Think of the experience and prestige to be gained, not only from serving the suffering poor, but from that considerable section of the community who would show just as keen a relish for cheap law as they now do for free medical services. If the members of the medical profession were paid for public services they could afford to be generous and contribute to the charities in the ordinary way, and would immensely gain in public estimation and influence. After all, the health of the community is the most important national asset. *Salus populi est suprema lex.* Let the nation pay for it, not the cinderella of the professions.

I have but little hesitation in adding a note on medical politics before the members of the Bradford Division, because this Division has already taken a prominent position in the British Medical Association on Medical Ethics, Medical Aid Societies, and the Cant about Commercialism in Medicine.

